# Study on the Superhydrophobic Properties of an Epoxy Resin-Hydrogenated Silicone Oil Bulk Material Prepared by Sol-Gel Methods

**DOI:** 10.3390/ma14040988

**Published:** 2021-02-19

**Authors:** Kui Zheng, Jie Zhu, Haifeng Liu, Xingquan Zhang, Enze Wang

**Affiliations:** 1 Research Center of Laser Fusion, China Academy of Engineering Physics, Mianyang 621010, China; 2 Analytical and Testing Center, Southwest University of Science and Technology, Mianyang 621010, China; liuhaifeng@swust.edu.cn (H.L.); zhangxingquan@swust.edu.cn (X.Z.); 3 School of Materials Science and Engineering, Southwest University of Science and Technology, Mianyang 621010, China; xnkjwang@163.com

**Keywords:** epoxy resin, sol-gel method, superhydrophobic property, contact angle, repairability

## Abstract

A superhydrophobic material was prepared by a simple and easily accessed sol-gel method using epoxy resin (E-51) and γ-aminopropyltriethoxysilane (KH-550) as the precursors, aqueous ammonia (NH_4_OH) as the catalyst and hydrogenated silicone oil (PMHS) as the hydrophobic modifier, and then pelleting the final product. The morphologies, surface chemical properties and thermal stability of the superhydrophobic bulk materials were characterized by scanning electron microscopy, Fourier infrared spectrometry and thermal analyzer. The hydrophobic properties and repairability of the as-prepared materials were also studied. The results showed that the prepared epoxy resin-hydrogenated silicone oil bulk materials were composed of tightly bound nanoparticles with a size of 50–100 nm in diameter. The material showed excellent superhydrophobic properties with a surface contact angle of 152°. The material also had good thermal resistance with a heat-resistant temperature of 300 °C and showed good repairability. The epoxy resin-hydrogenated silicone oil bulk superhydrophobic material exhibited excellent performance and showed wide application prospects.

## 1. Introduction

In nature, various insects and plants (e.g., water skipper, lotus leaf and rice) have water contact angles larger than 150°, thus exhibiting superhydrophobic properties [1]. These properties originate from the low surface energy of the coating layer and the dual micro-nano coarse structure. Due to their various unique properties such as self-healing, corrosion protection of metals, fluid resistance reduction and oil-water separation, the interfacial behavior of superhydrophobic materials has wide application prospects in the fields of self-cleaning, corrosion protection, snow resistance, fog resistance, low-temperature resistance, water surface motion drag reduction and oil-water separation [2,3,4,5]. Therefore, study on superhydrophobic materials has become a hot topic in the material research in recent years.

At present, the approaches to create superhydrophobic surfaces mainly focus on the construction of micro-nano dual structures and their modification with a low surface energy material through the use of sol-gel, vapor deposition, chemical etching, electrochemical and laser etching methods [6,7,8,9,10,11,12,13,14]. Rodriguez et al. prepared silicon oxide aerogels and xerogels by the sol-gel method using tetramethoxysilane and methyl trimethoxy silane as the precursors, which were then processed into a powder and adhered to a glass surface. It was found by studying the hydrophobic properties that the superhydrophobic effect of the gel powder was good, with the contact angle of 167–170°, and the anti-drag effect of the coating to laminar flow 20–30% [15]. Chang et al. used a sol-gel method and added poly (propylene glycol) (PPG) into a SiO_2_ precursor solution. The coating had a contact angle higher than 150° and had superhydrophobic self-cleaning properties [16]. Lu et al. constructed a micro-nano hierarchical structure on a Ti surface using an electrochemical etching method that was then modified with a fluorosilane to realize the superhydrophobic properties [17]. Peng et al. reported a series of studies on the superhydrophobic coating and made a remarkable breakthrough in durable superhydrophobic and highly oleophobic coatings [18,19]. Compared with other methods, sol-gel method is a mild material preparation method and has the advantages of simple operation, controllable process and low costs, making it popular among researchers. Currently, the preparation of superhydrophobic coating has achieved good results [20,21,22]. However, the use of many of the coatings in practical engineering applications is difficult, with multiple problems to be solved. For example, the high cost of the raw materials, complex preparation technology and the poor heat resistance as well as anti-corrosion properties of the coatings limit their application. Therefore, the development and preparation of the superhydrophobic coatings with excellent performance, simple technology, moderate costs and using environmentally friendly processes are important for research.

E-51 is one of the epoxy resins, which is one kind of thermosetting polymeric material with excellent properties of heat aging resistance, high strength and anti-corrosion, and has been widely applied in the coating industry, plastic industry, chemical engineering and national defense sectors [23]. Hydrogenated silicone oil (PMHS) is a transparent and liquid polymer. It has excellent hydrophobic properties and has been widely applied as a water-proofing additive in base materials such as textiles, paper, wood and glass [24].

In the present work, a superhydrophobic material was prepared using an epoxy resin and hydrogenated silicone oil as the raw materials by the sol-gel method, and the superhydrophobic bulk material was prepared by the direct pressing and further surface polishing process. The morphology, surface chemical properties and thermal stability of the superhydrophobic bulk material were characterized using scanning electron microscopy, Fourier infrared spectrometry and thermal analyzer. Next, the hydrophobic properties and repairability of the prepared material were studied.

## 2. Experimental Sections

### 2.1. Experimental Reagents

PMHS, industrial grade, was commercially supplied by Xin Si Hai Chemical Industry Co., Ltd., Xiangyang, Hubei, China. Dibutyltin dilaurate, analytically pure, was commercially supplied by Chendu Kelong Chemical Reagent Factory, Chengdu, Sichuan, China. Ammonia solution (NH_4_OH, 25.0–28.0%), analytically pure, was commercially supplied by Chengdu Kelong Chemical Reagent Factory, Chengdu, Sichuan, China. Absolute ethanol, analytically pure, was commercially supplied by Chengdu Kelong Chemical Reagent Factory, Chengdu, Sichuan, China. Epoxy resin (E-51, C_21_H_23_ClFNO_2_, CAS: 61788-97-4), industrial grade, was commercially supplied by Nantong Xingchen Synthetic Material Co., Ltd., Nantong, Jiangsu, China. γ-aminopropyltriethoxysilane (KH-550), analytically pure, was commercially supplied by Chengdu Kelong Chemical Reagent Factory, Chengdu, Sichuan, China. In addition, 320 mesh sandpaper (SKY Lark, Dongguan Golden Sun grinding Co., Ltd., Dongguan, Guangdong, China) was used.

### 2.2. Preparation of Superhydrophobic Material

**Figure 1:**
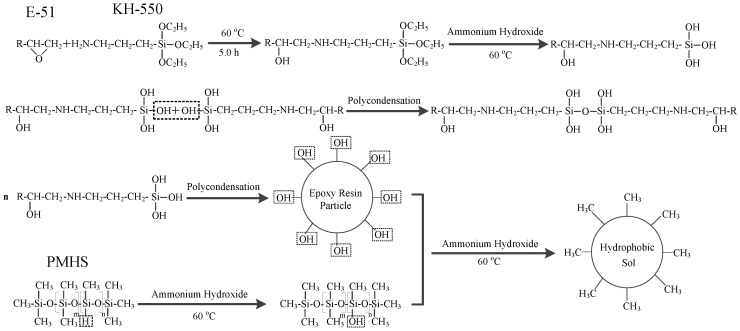
Synthetic reaction scheme of the hydrophobic gel.

First, 1.0 g of epoxy resin (E-51) was weighed, and 150 mL of absolute ethanol was added. The solution was dissolved using an ultrasonic bath and transferred into a three-neck flask. The solution was refluxed for 0.5 h, and then 1.5 mL of KH-550 was added. Open-loop modification was performed on the epoxy resin at a reaction temperature of 60 °C. After 5 h, 1.5 mL of PMHS, 3.0 mL of an ammonia solution and 2.0 mL of dibutyltin dilaurate were added. After 15 h, 15 mL of distilled water was added to the reaction solution. The superhydrophobic sol material was obtained after continued reaction for 24 h, as shown in the reaction scheme in Figure 1. The sol was placed in an oven and dried under air at 40 °C for 16 h to obtain the superhydrophobic material. The dried superhydrophobic material was milled into a powder and the powder was pressed into pellets using a pelletizer to obtain the bulk material (about 2 mm). The surface layer was removed with 320-mesh sandpaper to obtain the superhydrophobic block. The purpose of the polishing process was to construct the rough surfaces to increase hydrophobicity.

### 2.3. Material Characterization

The surface morphology of the superhydrophobic bulk material was observed using an Ultra 55 type field emission scanning electron microscope (Carl Zeiss AG, Jena, Germany). The sample was fixed on the conducting resin and gold-plated. FT-IR (Fourier transform infrared spectrometry) spectra were collected in the range of 4000–400 cm^−1^ by Spectrum One (Version BM) FT-IR (PerkinElmer, Waltham, MA, USA) spectrometer with 32 scans resolution of 2 cm^−1^. Approximately 10% (mass fraction) of the solid sample was mixed with spectroscopic grade KBr. The water contact angle of the bulk material was determined by a DSA30 type contact angle analyzer (Kruss, Hamburg, Germany). Next, 3 μL of deionized water was dropped on the sample surface using a micro-injector at room temperature. Five different sites were measured on each sample and the average value was calculated. Thermogravimetric analysis measurements were carried out using an STD Q 600 of TA instrument in nitrogen (N_2_) atmospheres under heating rates of 10 °C/min. A sample weight of about 10 mg was used for these measurements.

## 3. Results and Discussion

### 3.1. Analysis of Chemical Bond Characteristics

**Figure 2:**
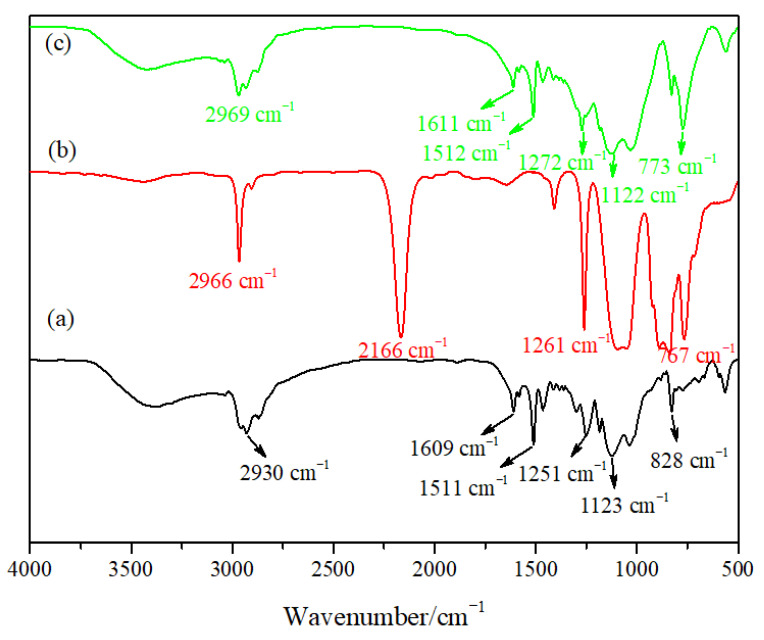
Infrared spectra of materials: (**a**) epoxy resin particles, (**b**) hydrogenated silicone oil (PMHS) and (**c**) hydrophobic gel.

Figure 2 shows the infrared spectra of the epoxy resin particles, PMHS and hydrophobic gel. Figure 2a is the infrared spectrum of the epoxy resin prepared using E-51 and KH-550 as the precursors and an ammonia solution as the catalyst. There was a strong absorption peak at 3369 cm^−1^, which may be ascribed to the Si–OH stretching vibration peak or remaining OH-groups after the epoxide ring opening, needing further study to confirm. The peak at 2930 cm^−1^ was the C–H stretching bending peak, and the peak located at 1609 cm^−1^ can be ascribed to the bending vibrations of H–OH. The peak at 1511 cm^−1^ was the bending vibration of the benzene ring, the peak at 1251 cm^−1^ was the bending vibration of C–O, the peak at 1123 cm^−1^ can be attributed to the stretching vibration of Si–O–Si [25], and the peak at 828 cm^−1^ was the stretching vibration of C=H. The peak at 2166 cm^−1^ in the infrared spectrum for PHMS can be ascribed to the vibration absorption peak of Si-H, the peak at 767 cm^−1^ was the vibration absorption peak of Si–CH_3_. It can be seen in Figure 2c that a new hydrophobic Si–CH_3_ group absorption peak appeared at 773 cm^−1^ in the hydrophobic gel. The appearance of the new peak indicated that a hydrophobic gel was successfully prepared. The intensity of the Si–O–Si antisymmetric stretching vibration peak at 1122 cm^−1^ obviously increased, which was due to the large number of Si–O–Si groups in the PMHS molecular structure. The intensity of the Si-OH absorption peak at 2969 cm^−1^ obviously decreased compared with that in the epoxy resin particles, indicating that a large amount of –OH was consumed during reaction, but some hydroxyl groups did not participate in the reaction.

### 3.2. Hydrophobicity Analysis

**Figure 3:**
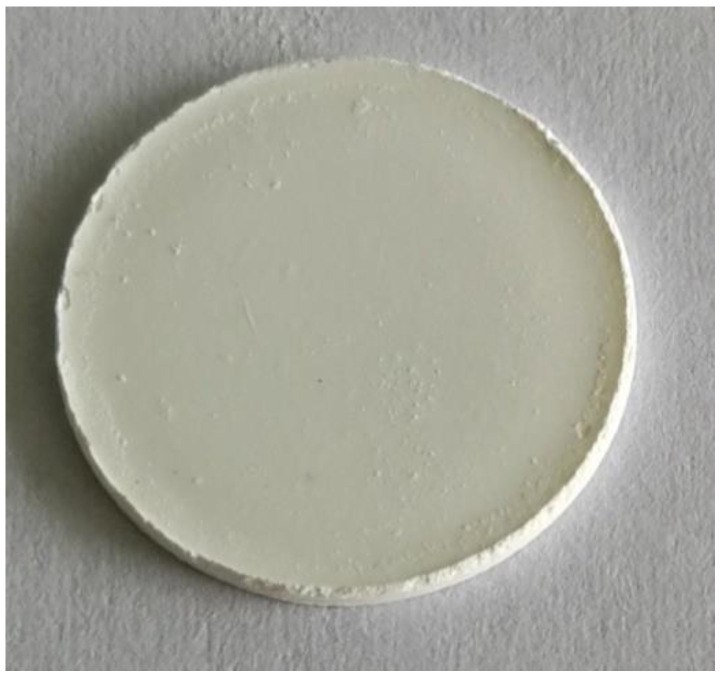
Superhydrophobic bulk material prepared by pelleting.

**Figure 4:**
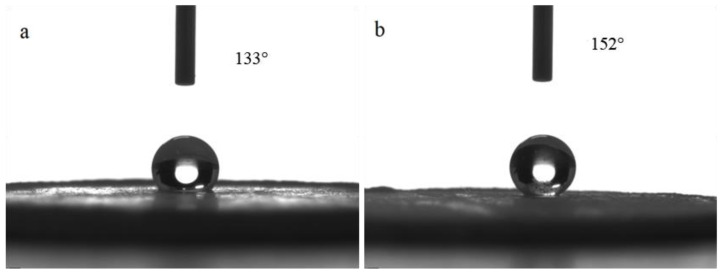
The influence of polishing on the contact angle of bulk material: (**a**) before polishing, (**b**) after polishing.

The surface morphology of the hydrophobic bulk material prepared by pelleting the reaction solution is shown in Figure 3. The contact angle measurement results are shown in Figure 4a. The surface of the pelleted bulk material was hydrophobic with a water contact angle of 133°. The contact angle sample after being polished with 320 mesh sandpaper is shown in Figure 4b. It can be seen that the surface of the bulk sample after being polished was superhydrophobic with a water contact angle of 152° and a roll angle of 8°.

**Figure 5:**
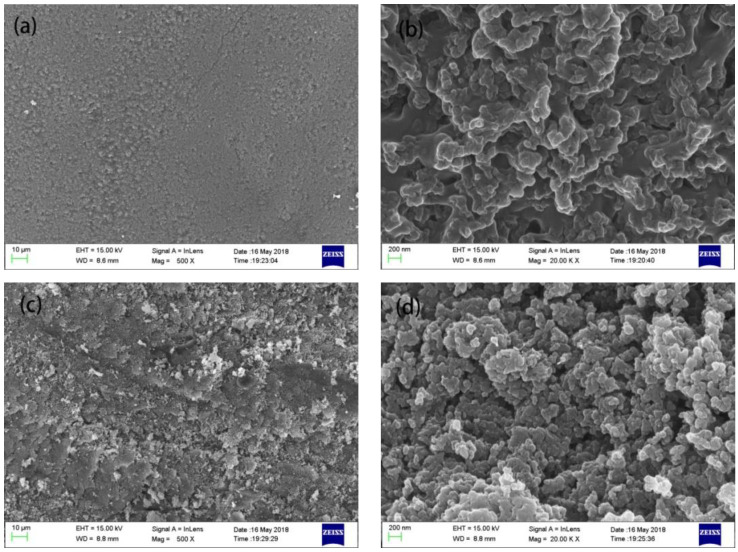
SEM images of the superhydrophobic bulk sample under different magnifications. (**a**): before polishing, 500×; (**b**): before polishing, 20,000×; (**c**): after polishing, 500×; (**d**): after polishing, 20,000×.

In order to explore the reason for the different hydrophobic properties of the samples, the morphology of bulk samples before and after polishing were observed, as shown in Figure 5. As seen in Figure 5a the pellet morphology before polishing was smooth and dense, which was due to the external compression during centrifugation. Nanoparticles with diameters ranging from 50–100 nm were found in the sample before polishing as shown in Figure 5b. The pores between the nanoparticles caused the surface to be coarse and did not meet the requirements for a superhydrophobic material as the contact angle was only 133°. Figure 5c shows the morphology of the polished sample under 500 times magnification, in which there are scratches on the surface. Figure 5d shows the micro-morphology of the sample under 20,000 times magnification. The nanoparticles on the material surface after polishing were around 50 nm in diameter and were tightly combined. The tightly packed nanoparticles formed the appropriate coarse structure on the micro-nano scales needed to create a superhydrophobic surface [26].

### 3.3. Thermal Properties

The superhydrophobic bulk material was heated at different temperatures for 2 h after the polishing step. After heat treatment, the contact angle and rolling angle of each sample were measured and the results are shown in Table 1.

**Figure 6:**
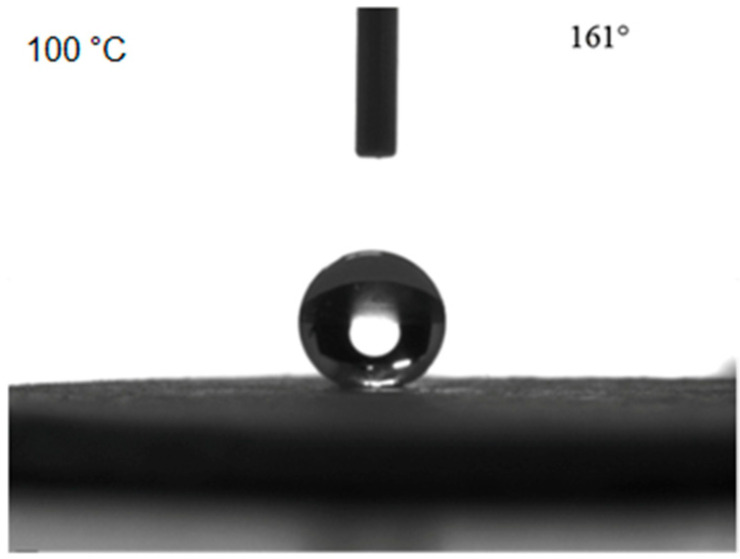
Contact angle measurement results for the bulk surface after 100 °C heat treatment.

The contact angle of the bulk sample first increased and then decreased, while the rolling angle first decreased and then increased with increasing heat treatment temperature. When the heat treatment temperature was 100 °C, the contact angle reached 161°. When the heat treatment temperature was lower than 100 °C, the contact angle increased with increasing temperature, which was because the heat treatment led to the disappearance of the structural water in the bulk material, and therefore, the hydrophobicity of the surface increased. When the temperature was higher than 100 °C, the increased heat treatment temperature might cause a decrease of the surface hydrophobicity of the bulk sample due to the thermal decomposition of the surface. The contact angle measurements at the surface of the bulk sample are shown in Figure 6 for a heat treatment temperature of 100 °C.

**Figure 7:**
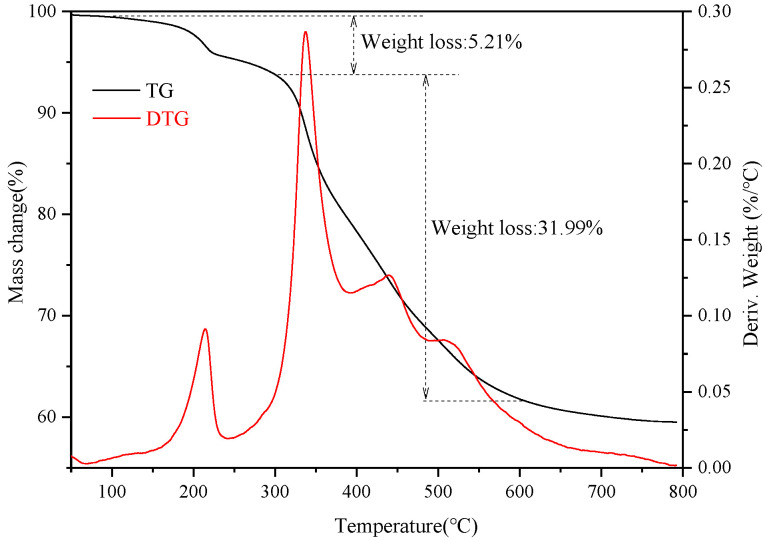
TG-DTG curve of the superhydrophobic bulk material.

Thermal analysis (TG) was conducted for the superhydrophobic bulk material after polishing. Figure 7 shows TG-DTG (derivative thermogravimetric analysis) curve of the superhydrophobic bulk material. It can be seen from the figure that when the temperature increased from room temperature to 150 °C, the weight of the material decreased slightly. When the temperature increased from 150 to 300 °C, the material showed a slight weight loss of 5.21%, which might be mainly due to the evaporation and decomposition of impurities in the material. When the temperature was further increased from 300 to 600 °C, the weight loss of the material was 31.99%, suggesting that there was decomposition of the bulk material. It can be seen from the TG curve that the hydrophobic material exhibited good thermal resistance properties, further verifying that higher heat treatment temperatures caused the material to decompose and thus decreased the surface hydrophobicity [27]. It can be ascertained by the TG that the prepared superhydrophobic material showed excellent thermal resistant performance and the thermal resistant temperature was as high as 300 °C

**Figure 8:**
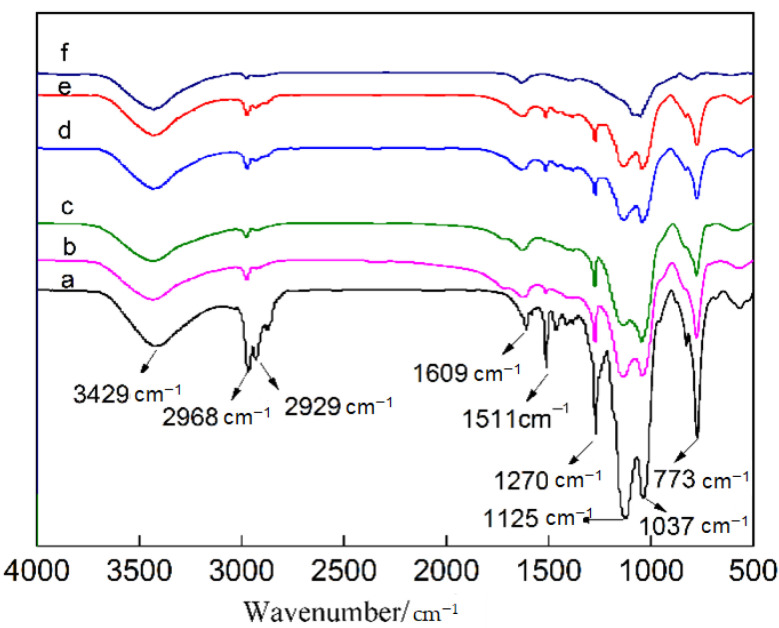
IR spectra analysis of the material treated at different heat treatment temperatures. (**a**) 50 °C; (**b**) 100 °C; (**c**) 150 °C; (**d**) 200 °C; (**e**) 250 °C; (**f**) 300 °C.

In order to further study the reasons for the differences in the surface hydrophobicity of the samples, infrared (IR) spectra were measured again after heat treatment at different temperatures, as shown in Figure 8. As seen in Figure 8a, the Si–OH stretching vibration absorption peak appeared at 3429 cm^−1^, C–H stretching vibration absorption peaks appeared at 2968 and 2929 cm^−1^. A weak H–OH bending vibration absorption peak was located at 1609 cm^−1^, a strong Si–O–Si stretching vibration and Si–CH_3_ absorption peak of the hydrophobic groups could be found at 1270 and 773 cm^−1^, respectively, and the antisymmetric Si–O–Si stretching vibrations were located at 1125 and 1037 cm^−1^. Comparing the IR spectra of the samples treated at different temperatures suggested that as the heat treatment temperature increased, the absorption peaks of the hydrophobic groups including C–H, Si–CH_3_ and Si–O–Si gradually weakened, which was an important reason for the weakened hydrophobicity at high heat treatment temperature.

### 3.4. Repairability

**Figure 9:**
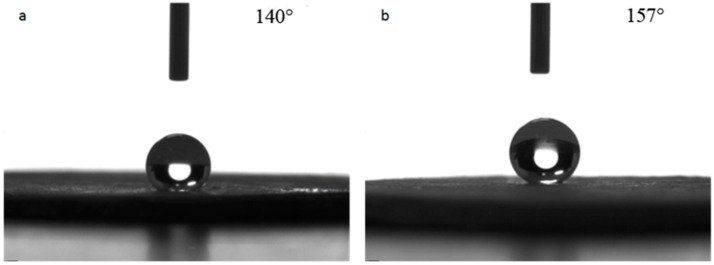
Repairability tests of hydrophobic property of the superhydrophobic bulks. (**a**): pressed bulk sample; (**b**): after being polished again.

The repairability of the superhydrophobic bulk sample treated at 100 °C was tested. The surface of the sample was pressed to damage the surface micro-structure and the contact angle was measured. Next, the surface was polished with 320-mesh sandpaper, and the contact angle was measured again to evaluate the repairability. The test results are shown in Figure 9. It can be seen from Figure 9a that the contact angle of the pressed bulk sample was 140°, which was close to the value before polishing, indicating that the pressing on the surface of the bulk sample damaged the surface micro-structure and thus reduced its hydrophobicity. When the pressed sample was polished again, the surface contact angle reached 157° as shown in Figure 9b, which indicated the bulk sample had good repairability, withstood environmental testing and possessed good practical value.

## 4. Conclusions

In summary, an epoxy resin-hydrogenated silicone oil hydrophobic material was easily prepared via the sol-gel method, and then the superhydrophobic bulk sample was prepared by the direct pressing and further surface polishing process. The material showed an excellent surface water contact angle of 152°. It also had good thermal resistance and repairability and was heat resistant up to a temperature of 300 °C. The results indicated that, due to the simple preparation process and excellent performance, the epoxy resin-hydrogenated silicone oil bulk superhydrophobic material has wide application prospects.

## Figures and Tables

**Table 1:** Influence of heat treatment temperature on the contact angle and rolling angle of the superhydrophobic bulk sample.

Heat Treatment Temperature (°C)	50	80	100	150	200	250	300
Contact Angle (°)	153 ± 2	158 ± 2	161 ± 2	156 ± 2	154 ± 2	153 ± 2	151 ± 2
Rolling Angle (°)	8	6	4	5	5	5	8

## Data Availability

Data sharing is not applicable.
